# Research on Sparse Denoising of Strong Earthquakes Early Warning Based on MEMS Accelerometers

**DOI:** 10.3390/mi13122113

**Published:** 2022-11-29

**Authors:** Jiening Xia, Zhigao Chen, Jiang Yang, Yuxiu Chen, Benyan Tan, Zongxuan Wu

**Affiliations:** 1 Hubei Key Laboratory of Earthquake Early Warning, Institute of Seismology, Wuhan 430071, China; 2 Hubei Earthquake Administration, Wuhan, 430071, China; 3 Wuhan Institute of Seismologic Instrument Co., LTD, Wuhan 430071, China

**Keywords:** MEMS accelerometers, earthquake early warning, sparse denoising, non-seismic interference, sparse classification

## Abstract

In view of the fact that the noise in the same frequency band as the useful signal in the MEMS acceleration sensor observation data cannot be effectively removed by traditional filtering methods, a denoising method for strong earthquake signals based on the theory of sparse representation and compressive sensing is proposed in this paper. This skillfully realized the separation of strong earthquake signals from noise by adopting a fixed dictionary and utilizing sparse characteristics. Furthermore, considering the weakness of the sparse denoising method based on the fixed dictionary in the high signal-to-noise ratio, a spare denoising method based on learning an over-complete dictionary is proposed. Through the initial given seismic data, the ideal over-complete dictionary is trained to achieve seismic data denoising. In addition, for the interference waves of non-seismic events, this paper proposes an idea based on sparse representation classification to remove such non-seismic interference directly. Combining the ideas of noise reduction and non-seismic event elimination, we can obtain a standard sparse anti-interference denoising model for earthquake early warning. It’s innovative that this model implements the sparse theory into the field of earthquake early warning. According to the experimental results, in the case of heavy noise, the denoising model based on sparse representation can reach average SNR of 8.73 and an average MSE of 29.53, and the denoising model based on compression perception can reach average SNR of 7.29 and an average MSE 41.34, and the denoising model based on learning dictionary can reach average SNR 11.07 and average MSE 17.32. The performance of these models is better than the traditional FIR filtering method (average SNR −0.73 and average MSE 260.37) or IIR filtering method (average SNR 4.73 and average MSE 73.95). On the other hand, the anti-interference method of the sparse classification proposed in this paper can accurately distinguish non-seismic interference events from natural earthquakes. The classification accuracy of the method based on the noise category of the selected test data set reaches 100% and achieves good results.

## 1. Introduction

Earthquakes are one of natural disasters that seriously threaten the safety of human life and property. Due to the extremely complicated process of earthquake origin and occurrence, reliable short-term earthquake prediction cannot be obtained at present [1,2]. However, after an earthquake occurs, if the P wave can be used to identify the final scale of the earthquake in the early stage of the fault rupture, the earthquake warning information will be sent to the early warning target area by electrical signals before the destructive S wave arrives. In this way, disaster mitigation measures can be taken in advance in areas receiving early warning information [3,4,5,6].

In recent years, the application of MEMS technology in early earthquake warning has become more and more popular. The application of MEMS accelerometers in seismic observation provides technical support for the dense seismic observation network and dense seismic array. The traditional P-wave-based earthquake early warning technology has “early warning blind spots” in principle, and the establishment of a dense earthquake observation network is expected to break through the early warning blind area. Due to the low cost, the distance between stations of a dense seismic observation network can be reduced from 10 km to 1 to 2 km or even hundreds of meters. In this case, the location of the first seismic station that observes the earthquake can be regarded as the epicenter (in line with the traditional seismic positioning class I accuracy), greatly speeding up the real-time nature of the early warning. However, due to the existence of noise, the rapid determination of basic parameters such as magnitude and the estimation of ground motion has serious interference. Therefore, the focus of this paper is the noise reduction in seismic observation data and the identification of non-seismic interference events.

Theoretical and experimental analysis shows that the noise of a strong earthquake early warning system based on MEMS accelerometers mainly comes from the self-noise of MEMS accelerometers themselves. When the epicenter is far away, the seismic wave amplitude is small and easily affected by noise. The signal-to-noise ratio of the observation data of strong motion has a direct impact on the accuracy of the early warning information. The signal-to-noise ratio of strong earthquake observation data is not only reduced by noise, but strong noise can also interfere with earthquake early warning systems and cause false alarms. For the earthquake early warning system, in addition to the noise superimposed on the earthquake events, the more complicated situation is the environmental noise impact of various non-seismic events. For example, environmental vibration interference waves such as passing vehicles, blasting, and dynamic compaction are still obvious to the early warning system even after such interference is filtered in the time domain.

Due to the ubiquitous presence of noise, the conventional signal processing method needs to be used first to reduce the noise of the observed signal [2,7,8,9,10,11]. Time-domain filtering is the most common noise reduction method for seismic data in earthquake early warning systems, which generally uses low-pass or band-pass filters, such as the FIR or IIR filter with the flattest amplitude-frequency response. The design of the time domain filter is simple and easy to implement, but the premise of effective denoising is that the frequency domain of the noise does not coincide with that of the signal. So, it is impracticable when the frequency domain of the noise and the signal coincide. On the other hand, time-domain filtering is also unfeasible when the signal-to-noise ratio is too low, and the noise is large.

In recent years, with the continuous development of computer technology, denoising based on sparse theory has been gradually paid attention to. As an effective signal expression, sparse signal representation is widely used in signal processing fields for its good robustness, strong generalization, and anti-interference ability. According to the development of sparse theory, the sparse representation theory first appeared, and then the compressive perception theory. Compressive sensing can be seen as an application of sparse representation, focusing on reconstructing observations by using sparse prior knowledge. Both of these involve the selection of dictionaries, which leads to a new direction of dictionary learning. In this paper, we will focus on earthquake early warning and study noise reduction methods for strong earthquake signals based on three theoretical branches: sparse representation, compressive sensing, and dictionary learning. A denoising method for strong earthquake signals based on the theory of sparse representation and compressive sensing is proposed in this paper. This skillfully realized the separation of strong earthquake signals from noise by adopting a fixed dictionary and utilizing sparse characteristics. Furthermore, considering the weakness of the sparse denoising method based on the fixed dictionary in the high signal-to-noise ratio, a spare denoising method based on learning an over-complete dictionary is proposed. Through the initial given seismic data, the ideal over-complete dictionary is trained to achieve seismic data denoising. According to the experimental results, In the case of light noise, the denoising model based on sparse representation can reach average SNR of 21.96 and an average MSE of 1.40, and the denoising model based on compression perception can reach average SNR of 17.77 and an average MSE 3.68, and the denoising model based on learning dictionary can reach average SNR 23.75 and average MSE 0.93. The performance of these models is better or similar to that of the traditional FIR filtering method (average SNR 0.87 and average MSE 179.94) or IIR filtering method (average SNR 18.41 and average MSE 3.17).

In the case of heavy noise, the denoising model based on sparse representation can reach average SNR of 8.73 and an average MSE of 29.53, and the denoising model based on compression perception can reach average SNR of 7.29 and an average MSE of 41.34, and the denoising model based on learning dictionary can reach average SNR 11.07 and average MSE 17.32. The performance of these models is better than the traditional FIR filtering method (average SNR −0.73 and average MSE 260.37) or IIR filtering method (average SNR 4.73 and average MSE 73.95).

Furthermore, for earthquake early warning systems, in addition to the noise superimposed on earthquake events, the environmental noise impact of various non-seismic events is more complicated. For example, for environmental vibration interference waves such as passing interference, blasting, dynamic compaction, and so on, after they pass the time-domain filtering, the interference to the early warning system is still obvious. At present, there are few types of research on this issue at home and abroad, and few related studies have positively considered the problem of anti-interference and anti-false triggering. Usually, most the studies are only in the trade-off between false and missed alarms. Therefore, this paper proposes a based on the sparse classification to directly eliminate such non-seismic event interference. According to the experimental results, the classification accuracy of the sparse classification anti-interference method based on the noise category of the selected test data set reaches 100%, achieving good results.

## 2. Sparse Theory and Noise Reduction

### 2.1. Noise Reduction Based on Sparse Representation

Generally, signal representation is to represent the original signal by a certain transformation. Thus, the original signal is projected into the transform domain. The N-dimensional discrete time domain signal x can be represented by a set of orthogonal basis linear combinations:

(1) $$x=\sum_{i=1}^{N}\alpha_{i}\varphi_{i}\;\mathrm{or}\;x=\Phi \alpha$$

Among them, $\Phi =[\varphi_{1}\left|\varphi_{2}\right|\ldots |\varphi_{N}]$ is an orthogonal dictionary composed of a set of orthogonal bases, $\varphi_{i}\in R^{N\times 1}$ is the basis vector or dictionary atom. $\alpha =[\alpha_{1}\left|\alpha_{2}\right|\ldots |\alpha_{N}]$^T^ is the representation coefficient of x in the dictionary. According to the definition of signal sparsity, if most of the elements in the representation coefficient α are zero and few are non-zero, the representation coefficient α can be considered sparse, and signal x is also sparse in the basis of the group.

In practical applications, for $x\in R^{N\times 1}$, a specific set of orthogonal bases cannot adapt to all signals, so it’s difficult for the given dictionary $\Phi \in R^{N\times N}$ to sparsely represent all signals. Therefore, a set of over-complete base vectors $D=[d_{1}\left|d_{2}\right|\ldots |d_{M}]$, $d_{i}\in R^{N\times 1}$, M > N, is needed. At this time, dictionary D is called an over-complete dictionary or redundant dictionary, and the signal under over-complete dictionary D is expressed as:

(2) $$x=D\alpha =\overset{N}{\underset{i=1}{\sum }}\alpha_{i}d_{i}$$

Formula (2) is an underdetermined system of equations, in many application problems, constraints can be added to make it the only optimal solution if the conditions are met.In the framework of constraints, the sparse representation problem is described as:

(3) $$P_{0}:{\min\|\alpha \|}_{0}\;s.t.x=D\alpha \;$$

If there are noises in the signal x, the mathematical model of the signal with additive noise can be described as:

(4) $$x=x_{0}+e\;$$

$x_{0}$ is defined as an ideal signal without noise and e as additive noise. By substituting the signal x into the (3) model, we can obtain:

(5) $$P_{0}^{*}:{\min\|\alpha \|}_{0}\;s.t.\|x-{D\alpha \|}_{2}\leq \varepsilon \;$$

ε is a very small positive number representing the maximum allowable error.

The signal sparse decomposition problem described by Formula (5) is a minimization problem based on 0-norms. It is a difficult NP problem that is very difficult to solve directly. Donoho, Tao et al. proved that when the required representation coefficient is sparse enough, solving the 0-norm optimal problem is equivalent to solving the 1-norm optimal problem. That is, the same result can be obtained [12,13,14,15].

For the signal with addictive noise, it can be described as:

(6) $$P_{1}^{*}:{\min\|\alpha \|}_{1}\;s.t.\|x-{D\alpha \|}_{2}\leq \varepsilon \;$$

We now know that if certain conditions are met for the problem $P_{1}$, the unique sparse solution can be obtained. So, what are the common methods for such problems? The greedy algorithm is one of them. The idea of the algorithm is also to select a local optimization solution by iteration, hoping to select the best atom representing the signal to approach the original signal step by step [16]. Subsequently, based on the MP algorithm, several improved algorithms have been proposed, such as the Orthogonal Matching Pursuit (OMP) algorithm [17].

The relaxation algorithm is another type of optimization for the 1-norm [18]. The 1-norm of the representation coefficient is used to measure its sparseness. Through the minimization of the 1-norm of the representation coefficient, the sparse representation signal problem is described as a type of constrained optimization problem, that is, the $P_{1}$ problem, and then linear programming is used to solve the problem.In the past few decades, scholars have performed lots of research work to solve linear programming problems and have proposed methods such as simplex, interior-point-methods, gradient descent [19], iterative projection algorithm [20], minimum regression algorithm [21], gradient projection algorithm [22].

**Figure 1:**
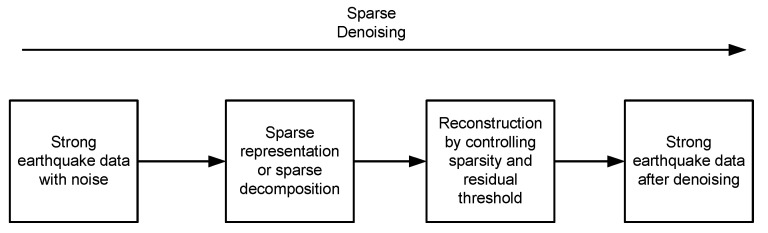
Process Flow of Strong Earthquake Data Denoising Based on Sparse Representation.

For earthquake early warning application, the general process flow of denoising based on sparse representation is shown in Figure 1.

The basic idea of denoising based on sparse representation is after sparse transformation on the strong earthquake data, parts of the effective signals in the transform domain correspond to the representation coefficients with larger amplitude, while the noises correspond to the representation coefficients with smaller amplitude. Therefore, if the representation coefficient of the strong earthquake data in this transform domain is sparser, that is, only a small number of coefficients with large amplitude, most of them are with very small amplitude, then the smaller coefficients of these noises are filtered out in the transform domain, and the strong earthquake data under the suppression of noises can be obtained after reconstruction. The noise reduction problem of strong earthquake data can be described in the following general form [22]:

(7) $$\{\begin{array}{c} \hat{\alpha }=\mathrm{argmin}{\left\|\alpha \right\|}_{1},\;s.t.\|x-{D\alpha \|}_{2}^{2}\leq \varepsilon \\ \hat{x}=D\hat{\alpha } \end{array}$$

D is an over-complete dictionary representing strong earthquake data. It can be a selected fixed transform dictionary (Discrete Cosine Transform, Discrete Fourier Transform, Wavelet Transform, etc.), or it can be an adaptive learning dictionary obtained from the data itself. The greedy algorithm or relaxation algorithm can be used for reconstruction, but there are some transformations. Due to the addition of noise, it’s hoped that the reconstructed signal does not contain noise but rather completely restores the noisy signal. The OMP algorithm will be mainly introduced. In order to distinguish it from the general OMP algorithm, the OMP algorithm for denoising is called the OMPDN algorithm. The main content of the algorithm is as follows:

(1) Input parameters: overcomplete dictionary D, strong earthquake signal x, sparsity K;
(2) Output parameters: sparse coefficient A of the strong earthquake signal, residual res;
(3) Initialization: residual res = x, index set initialization, number of iterations i = 1;
(4) Iterative Step 1: Calculate the inner product of the residuals and each atom (column vector) in the dictionary D;
(5) Iterative Step 2: Find the corresponding position of the maximum projection coefficient (internal product). If the maximum value is more than one, take the first one. Take the position as the i-th position of the index set;
(6) Iterative Step 3: Multiply the strong earthquake signal x with the pseudo-inverse matrix, which is composed of columns corresponding to the current index set in the dictionary D, to obtain the sparsity coefficient a;
(7) Iterative Step 4: Calculate the residual res. Stop the iteration if res is less than the set threshold or if the number of iterations reaches K. Otherwise, turn to step 1 and continue the iteration. When stopped, output sparse coefficient A, index, and residual.

### 2.2. Noise Reduction Based onCompressive Sensing

Compressive sensing theory is proposed after sparse representation. Tao, Candes, Donoho, et al. constructed a theoretical framework for compressive sensing [23]. Later, the theory received great attention in the fields such as information theory, medical imaging, optical radar imaging, image signal processing, pattern recognition, wireless communication, and geological exploration [24,25,26,27,28,29,30].

As mentioned earlier, in the theoretical framework of sparse representation, a one-dimensional vector x of length N can be represented by a set of linear combinations of standard orthogonal bases, as shown in Formula (1). In reverse, if a measurement matrix $\Phi \in R^{M\times N}\left(M\ll N\right)$ and a signal x are known, the linear measurement value y $\in R^{M\times 1}$ of the signal under the measurement matrix can be obtained,

(8) y=Φx 

Since dimension M of the measured value y is much smaller than dimension N of signal x, the purpose of compression is achieved. Similarly, the compressive sensing theory can be used to recover a high-resolution signal from a few observations. Obviously, when y and Φ are known, the next step is to consider how to reconstruct the sequence x. If the original signal x is K-sparse and y and Φ meet certain conditions, the theory proves that the signal x can be accurately reconstructed by solving the optimal norm $L_{1}$ problem of the measured value y, which can be described as:

(9) $$\hat{x}=\mathrm{argmin}\|x{\|}_{1}\;s.t.\;\Phi x=y\;$$

Similar to the problem in Section 2.1, the reconstruction problem of compressive sensing can also be solved by the greedy algorithm and relaxation algorithm.

The compressive sensing theory has also gone through several stages. The original models in Candes’ and Donoho’s articles were directly aimed at sparse signals, such as Formula (8), which discussed what kind of observation matrix and how to recover sparse signals from observed signals. Later, it was found that many signals x are not sparse in practical applications. $\alpha =\;\Psi^{\prime }\;x$ are obtained by a certain orthogonal transformation $\Psi^{\prime }$, where α is sparse and $\Psi^{\prime }$ is the transpose of Ψ; although x is not sparse here, x can be transformed into α through $\Psi^{\prime }$, then α coincides with the compressive sensing model as:

(10) $$y=\Phi \;\alpha \;\mathrm{or}\;y=\Phi \;\Psi^{\prime }\;x$$

Therefore, research on sparse transformation is the key point to this problem. This theoretical model is called the analysis model.

Later, researchers in many fields advocated using over-complete dictionaries to represent signals. For signals that are not sparse, they can be represented by over-complete dictionaries and sparse matrices. Candes gives a representation of compressed sensing in an over-complete dictionary. It is called the comprehensive model, that is:

(11) y=Φx=ΦDα

**Figure 2:**
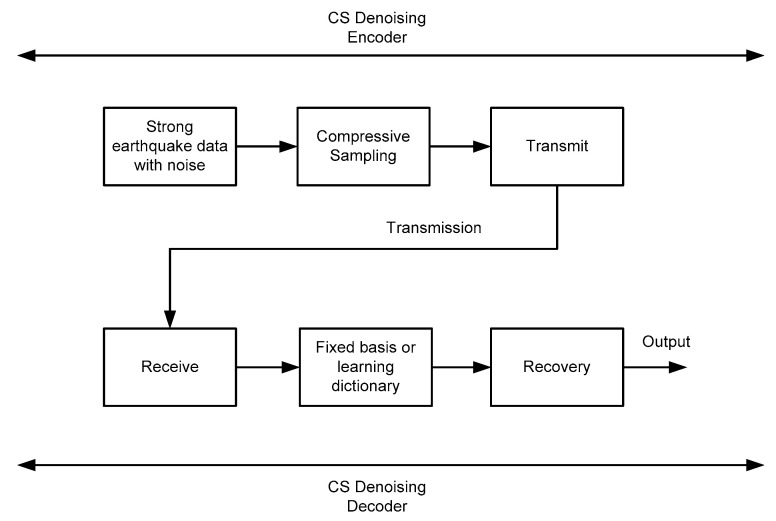
Process Flow of Strong Earthquake Data Denoising Based on Compressive Sensing.

For earthquake early warning application, the general process flow of denoising based on compressive sensing is shown in Figure 2.

Because of the addition of noise, the traditional BP algorithm doesn’t work, and the BPDN algorithm is applied. Suppose the pure strong earthquake signal is $x_{0}$, e is noise, and the noisy, strong earthquake signal is $x=x_{0}+e$. First of all, we need to derive a model under the framework of compressive sensing. From Formula (10), we can obtain:

(12) $$\begin{array}{l} \alpha =\;\Psi^{\prime }\;x\\ y=\Phi \alpha =\Phi \;\Psi^{\prime }\;x=\Phi \;\Psi^{\prime }(x_{0}+e)={\Phi \;\Psi^{\prime }\;x}_{0}+z \end{array}$$

For the analysis model of compressive sensing, the idea of denoising is more direct because the Φ and Ψ are fixed, but noises are mixed in the strong earthquake signal x. The main components of the signal are restored in the reconstruction algorithm by controlling the sparsity and residual threshold, and the non-main components and the noise-containing parts are discarded.

For the comprehensive models, as shown in Formula (11), the noise e is hidden in the dictionary D, and the sparse coefficient α, according to the BPDN algorithm, transforms the sparse decomposition of the noisy signal into the following optimization problems [22]:

(13) $${\min\|\alpha \|}_{1}\;s.t.\;{\left\|y-\Phi D\alpha \right\|}_{2}\leq \varepsilon$$

D is an over-complete dictionary, α is the sparse representation vector of y on the dictionary D, and ε>0 is the error allowable value.

### 2.3. Noise Reduction Based onLearning Dictionary

As mentioned earlier, there is the concept of the dictionary in both sparse representation and compressed sensing. Before the concept appeared, a single base atom was often used to approximate any signal. For example, in the Fourier transform, the direction and scale of the base are fixed. However, in the Wavelet Transform appeared the concept of multi-scale and multi-direction. The base became increasingly diversified so that more signals could be approached. Subsequently, the direction and shape of the base became more diversified. When it comes to the multi-scale geometric analysis, there is larger selectivity of the base and more shapes of the base atom. For example, the number of the direction of curves, brushes, and bands increased from the original three. In addition, more signals can be approached, and the base atom becomes more flexible.

However, no matter how many shapes the basis is, it is still fixed. It is still not enough to approximate the signal with a basis of a single shape. Even though a redundant dictionary composed of multiple bases can approximate a complex signal composed of multiple signals with lots of signal shapes in nature, it is impossible to approximate all signals with fixed-shape base atoms. So, people gradually realized that it is very important to learn the shape of atoms adaptively. Later, the concept of dictionary learning was gradually formed.

For Formulas (6) and (13), the learning dictionary can be used completely instead of the fixed dictionary D for sparse computation. The task of dictionary learning is to design a dictionary for a given class of signal training samples so that such signals can be sparsely represented in the dictionary. Dictionary learning can be understood as the capture of the characteristics of a certain type of signal, and each atom of the dictionary is a feature.

K-SVD is a classical dictionary learning algorithm [31,32,33,34,35]. The problem model solved by K-SVD, one of the most famous dictionaries learning algorithms, is:

(14) $$\underset{D,A}{\min}\|X-{\mathrm{DA}\|}_{F\;}^{2}\;\mathrm{subject}\;\mathrm{to}\forall i,{\|\;\alpha }_{i}\|{\;}_{0}\leq K$$

$X\in R^{M\times L}$ is a training sample matrix, each column of which is a training sample; $D\in R^{M\times N}$ is a dictionary to be learned; $A\in R^{N\times L}$ is a matrix composed of sparse vectors whose i-th column is $\alpha_{i}$; K is a sparse constraint. In the above problem, it is difficult to optimize the dictionary D and the sparse coefficient A at the same time, so an iterative step is adopted in the algorithm, with two operations for each iteration:

(1) Sparse coding: For the given dictionary D, solve the sparse representation A;
(2) Dictionary update: For the given sparse representation A, optimize the dictionary D.

In the sparse encoding step, the OMP algorithm is used in K-SVD, and in the dictionary update step, K-SVD updates the dictionary atom by atom. Specifically, we expect to minimize the following Formula (15) when updating the atom in column n:

(15) $$\|X-{\mathrm{DA}\|}_{F}^{2}={\left\|X-\overset{N}{\underset{j=1}{\sum }}d_{j}\alpha_{j}\right\|}_{F}^{2}={\left\|(X-\overset{N}{\underset{j\neq n}{\sum }}d_{j}\alpha_{j})-d_{n}\alpha_{n}\right\|}_{F}^{2}={\left\|E_{n}-d_{n}\alpha_{n}\right\|}_{F}^{2}$$

$\alpha_{j}$ represents the j-th vector of matrix A. When updating $d_{n}$, the other atoms are fixed. The idea of the K-SVD algorithm is to perform the SVD of $E_{n}$ in the Formula (15), and then update $d_{n}$ with the column vector corresponding to the maximum singular value. The specific algorithm flow is as follows:

(1) Input: training sample set X, initial dictionary $D_{0}$, sparse constraint K or error constraint ε or maximum number of iterations
(2) Output: sparse representation A, dictionary D
(3) Loop iteration of the following steps
(4) Sparse coding: For the specified dictionary ($D_{0}$ for the first time) of each iteration, use the OMP algorithm to solve the sparse coefficient
(5) Update dictionary atoms $d_{n}$ in order, i.e.
  For n = 1, 2, …, m
  (a) Label all training samples using atoms to obtain the matrix.
    $$\omega_{n}=\left\{i|1\leq i\leq M,{\;\alpha }_{n}\left(i\right)\neq 0\right\}$$
  (b) Calculate the error matrix, i.e.,
    $$E_{n}=X-\sum_{j\neq n}^{N}d_{j}\alpha_{j}$$
  (c) Select the error sub-matrix $E_{n}^{R}$ based on the matrix $\omega_{n}$, i.e.,
    $$E_{n}^{R}=E_{n}\left(:,\omega_{n}\right)$$
  (d) Perform singular value decomposition SVD, i.e.,
    $$E_{n}^{R}=U\Delta V^{T}$$
  (e) Update atom $d_{n}$, i.e.,
    $$d_{n}=U\left(:,1\right)$$
  (f) Update the sparse coefficient of the corresponding position $d_{n}$, i.e.,
    $$\alpha_{n}^{\prime }\left(\omega_{n}\right)=\alpha_{n}^{R}=\Delta \left(1,1\right)V\left(:,1\right)$$
    End for
(6) If the specified number of iterations or $\|X-{\mathrm{DA}\|}_{F}^{2}<\varepsilon^{2}$ is reached, the iteration is terminated. Otherwise, execution continues.

## 3. Classifying Anti-Interference Method Based on Sparse Representation

As mentioned above, the denoising method based on sparse representation can be used for denoising of strong earthquake data. However, some non-seismic events, such as passing interference, blasting, dynamic compaction, and other man-made environmental vibration interference waves, do not contain real seismic information at all. Even with sparse denoising methods, no valid data can be obtained. At present, there are few positive solutions to such problems, usually only a trade-off between false and missed alarms.This paper proposes a classifying anti-interference method based on sparse representation, which identifies seismic events and non-seismic events through the idea of classification, thereby eliminating complex environmental vibration interference.

The main idea of this method is to collect enough non-seismic event interference waves (assuming a total of i categories) to form a training set, defined as dictionary D. For a given test sample x belonging to the class i, sparse representation $\hat{\alpha }$ can be obtained by Formula (6). Ideally, it is estimated that the non-zero elements of $\hat{\alpha }$ only exist at positions corresponding to class i in D, so it is easy to determine the belonging category of x [36,37,38,39,40,41,42]. The specific method and process are as follows:

For each class i, let $\delta_{i}:R^{n}\to R^{n}$ be the characteristic function that selects the coefficients related to class i. In addition, for $\alpha \in R^{n}$, the non-zero elements in vector $\delta_{i}\left(\alpha \right)\in R^{n}$ are the elements in α that are related to class i. Using only the elements related to class i, we can write the estimated value of the test sample x as ${\hat{x}}_{i}={D\delta }_{i}\left(\hat{\alpha }\right)$, calculate all the differences between ${\hat{x}}_{i}$ and x, and attribute x to the class that minimizes the residual (Wright et al. 2008)[36]:

(16) $${\min\;r}_{i}\left(x\right)=\|x-{D\delta }_{i}{\left(\hat{\alpha })\right\|}_{2}$$

The sparse representation classifying algorithm is as follows:

(1) Input: training matrix $D=\left[d_{1},d_{2},\ldots ,d_{k}\right]\in R^{m\times n}$ of class k, test samples $x\in R^{m}$ (optional error tolerance ε>0)
(2) Normalize all column vectors in D.
(3) Solve the minimum $l_{1}$ norm problem:
  $$\begin{array}{c} \hat{\alpha }={\mathrm{argmin}\|\alpha \|}_{1}\;s.t.\;D\alpha =x\\ (\mathrm{or}\;\hat{\alpha }={\mathrm{argmin}\alpha }_{1}\;s.t.\;\|x-{D\alpha \|}_{2}\leq \varepsilon \end{array}$$
(4) For i=1⋯k, calculate the reconstruction residual $r_{i}\left(x\right)=\|x-{D\delta }_{i}{\left(\hat{\alpha })\right\|}_{2}$
(5) Output: $\mathrm{identity}\left(x\right)={\arg\;\mathrm{minr}}_{i}\left(x\right)$.

## 4. Experiment

### 4.1. Data Preparation

**Figure 3:**
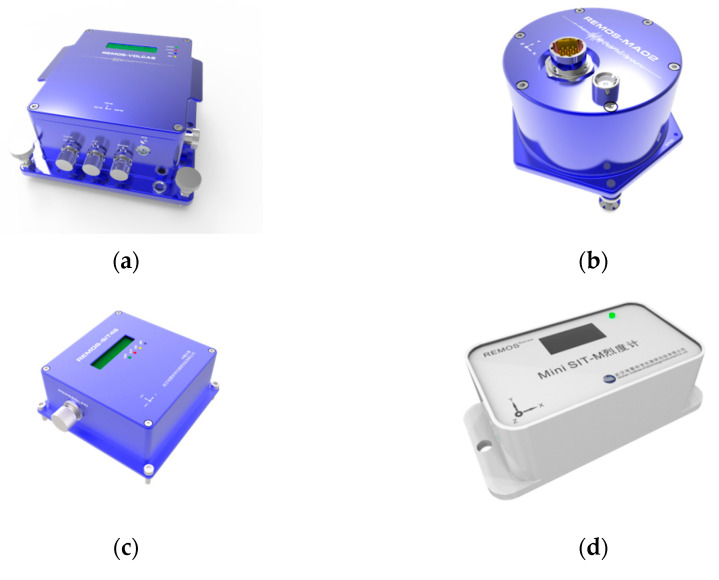

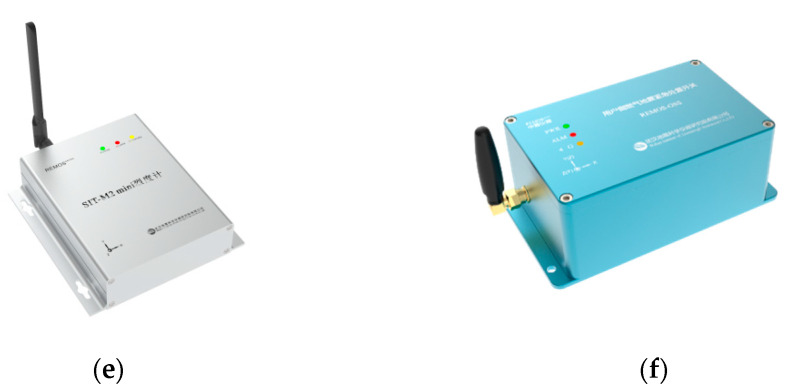
The strong earthquake monitoring equipment (**a**) is the integrated strong motion seismograph; (**b**) is the MEMS triaxial accelerometer; (**c**) is the high precision intensity meter; (**d**) is the first-generation simple intensity meter; (**e**) is the second-generation simple intensity meter; (**f**) is the seismic gas switch.

We have developed many strong earthquake monitoring instruments based on MEMS acceleration accelerometers, as shown in Figure 3, which have been widely used in professional seismic networks and the seismic monitoring field of major projects because of the cost advantage. MEMS acceleration sensor has a lot of noise due to their own process, including shot noise, thermal noise, flicker noise, etc. The noise outside the effective frequency band can be easily removed by filtering, but it is powerless over the noise inside the effective frequency band. To prove this, Section 4.2, 4.3, and 4.4 use traditional filtering methods to compare with the sparse denoising model proposed in this paper. The three traditional filters include FIR filters, Butterworth filters, and Chebyshev filters that belong to the same IIR filter. The three traditional filters are all configured as low-pass filters, and unified design parameters are adopted. The sampling rate is 200 Hz, the attenuation starts at 60 Hz, and the attenuation reaches 80 dB at 100 Hz.

The essence of denoising is not that the more signals filtered, the better because useful signals may also be filtered. In order to accurately compare and evaluate the effectiveness of various denoising methods, this paper uses reverse thinking, takes the original acceleration record obtained under the condition of extremely high signal-to-noise ratio as the basic signal, and compares the matching degree between the results of various denoising methods and the basic signal by adding noise, so as to evaluate the performance of various denoising methods.

The intensity of the noise is controlled by the variance sigma. In subsequent experiments, the intensity of the noise will be increased by increasing the sigma to further test the performance of the sparse filtering method. The signal-to-noise ratio (SNR) and mean square error (MSE) were used to measure the filtering effect. The larger the SNR value, the better the denoising effect. On the contrary, the smaller the MSE value, the better the denoising effect.

(17) $$\mathrm{SNR}=10\log\left(\frac{\sum_{i=1}^{N}x_{i}^{2}}{\sum_{i=1}^{N}{\left(x_{i}-{\hat{x}}_{i}\right)}^{2}}\right)=10\log\left(\frac{{\left\|x_{i}\right\|}_{2}^{2}}{{\left\|x_{i}-{\hat{x}}_{i}\right\|}_{2}^{2}}\right)$$

(18) $$\mathrm{MSE}=\frac{\sum_{i=1}^{N}{\left(x_{i}-{\hat{x}}_{i}\right)}^{2}}{N}=\frac{{\left\|x_{i}-{\hat{x}}_{i}\right\|}_{2}^{2}}{N}$$

N is the number of strong earthquake data; $x_{i}$, a good record of real strong motion with a large signal amplitude and extremely high signal-to-noise ratio, is the basic signal and ${\hat{x}}_{i}$ is the filtered signal.

Section 4.5 uses sine wave, square wave, and other simulated interference data, passing interference, blasting, dynamic compaction, and other real non-seismic event interference data and real strong earthquake record data to test the proposed method of classifying anti-interference.

### 4.2. Experiment of Noise Reduction Method Based on Sparse Representation

Based on the relevant content in Section 2.1, this experiment uses a DCT (Discrete Cosine Transform) dictionary and a DFT (Discrete Fourier Transform) dictionary. Define the data length N = 1024 and select one frame of strong earthquake data with a high signal-to-noise ratio and obvious seismic characteristics; the reconstruction algorithm uses the OMP algorithm and alternates the fixed dictionary for the experiment.

**Figure 4:**
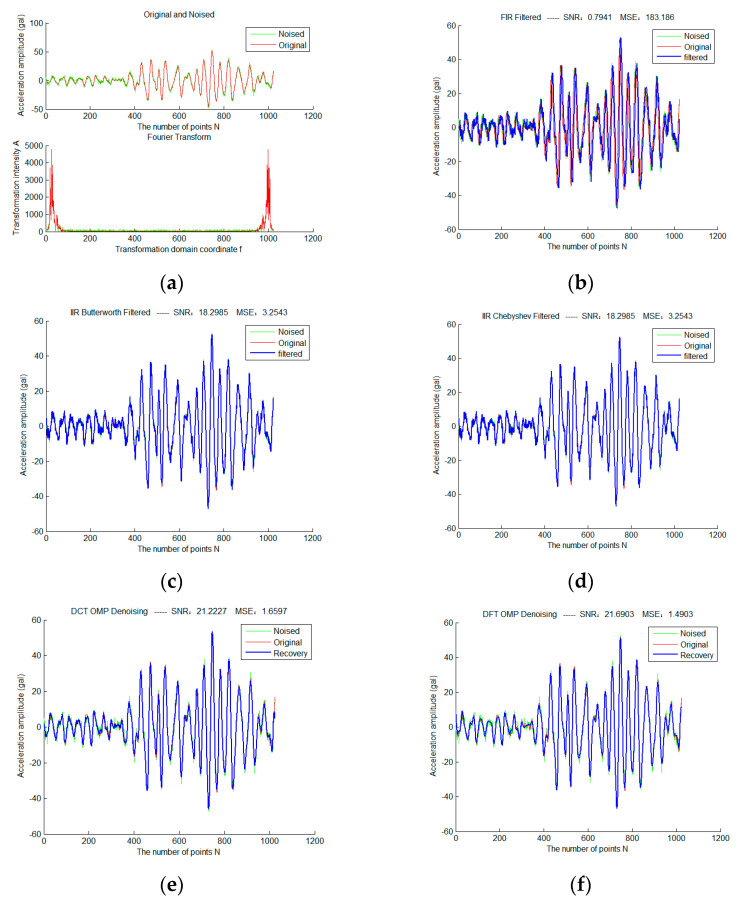
Sparse Denoising Experiment of a Frame of Strong Earthquake Signals Based on Different Fixed Dictionary (**a**) is the original and noise-adding waveforms of the frame signal in the time and frequency domain (sigma = 2); (**b**) is the result based on FIR filtering; (**c**) is the result based on Butterworth filtering; (**d**) is the result based on Chebyshev filtering; (**e**) is the OMP sparse denoising result based on the DCT dictionary; (**f**) is the OMP sparse denoising result based on the DFT dictionary.

First, add Gaussian white noise with sigma = 2 to a selected frame of strong earthquake data, and use three traditional filtering algorithms and sparse representation methods of different fixed dictionaries, as shown in Figure 4. Red is the original waveform, green is the waveform after adding noise, and blue is the filtered waveform. The colors and expressions used in all subsequent experimental results of the same type have the same meaning.

It can be seen that the total SNR of FIR filter in traditional filtering methods is very low due to serious phase lag. Therefore, in the subsequent experiments, we mainly discuss the IIR filter. Butterworth and Chebyshev filters obtain exactly the same filtering results due to the identical parameter configuration. The OMP sparse denoising results based on the DCT dictionary and DFT dictionary are significantly better than that based on traditional filtering. In addition, the sparse denoising model does not have the problem of phase lag.

**Figure 5:**
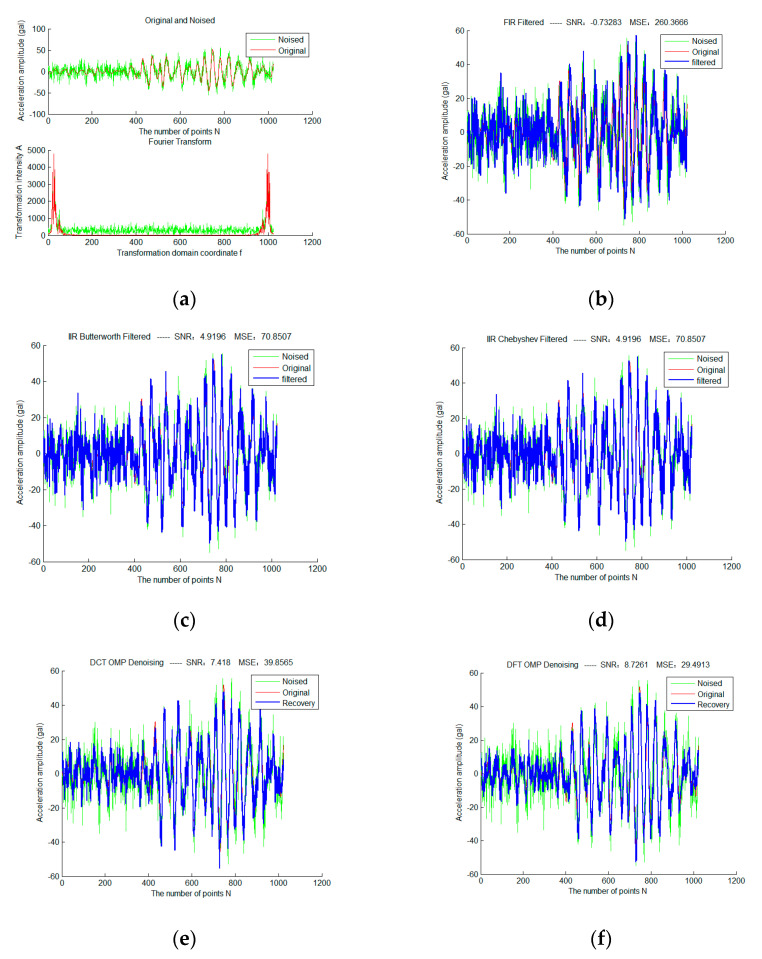
The Spare Denoising Experiment of a Frame of Strong Earthquake Signals Based on Different Fixed Dictionary (**a**) is the original and noise-adding waveforms of the frame signal in the time and frequency domain(sigma = 10); (**b**) is the result based on FIR filtering; (**c**) is the result based on Butterworth filtering; (**d**) is the result based on Chebyshev filtering; (**e**) is the OMP sparse representation denoising result based on the DCT dictionary; (**f**) is the OMP sparse representation denoising result based on the DFT dictionary.

Then, increase the sigma of the noise to 10, and repeat the above experiment. The result is shown in Figure 5.

It can be seen from Figure 5 that when the sigma of noise increases to 10, the effects of IIR filtering and sparse filtering decrease, but the results of the sparse denoising method are still significantly better than that of IIR filtering, which is equivalent to the results when the sigma is 2.

**Figure 6:**
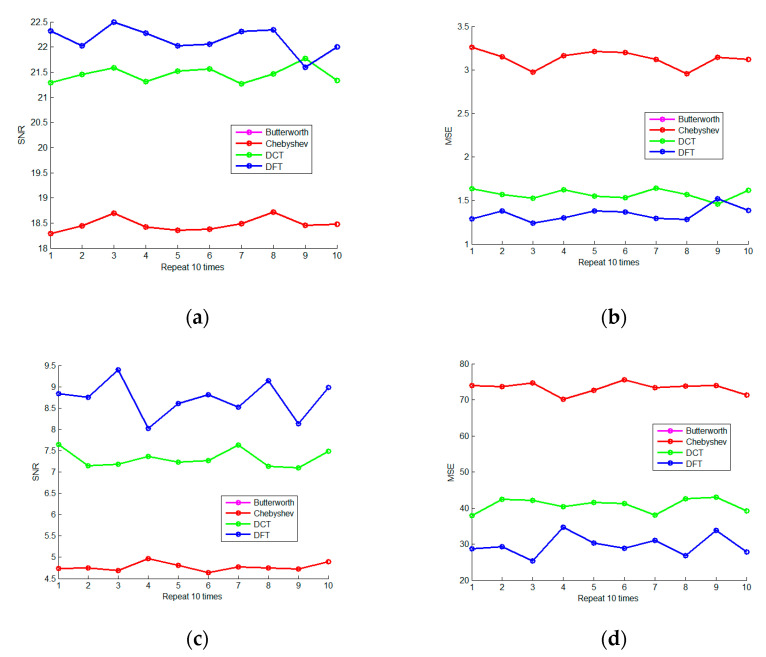
The Repeated Denoising Experiment (**a**) is the 10-times repetitive SNR curve of four denoising methods when sigma is 2; (**b**) is the 10-times repetitive MSE curve of four denoising methods when sigma is 2; (**c**) is the 10-times repetitive SNR curve of four denoising methods when sigma is 10; (**d**) is the 10-times repetitive MSE curve of four denoising methods when sigma is 10.

In order to examine the stability of the spare representation denoising method, we set the sigma to 2 and 10 and separately repeated the denoising experiments 10 times. In addition, the results are shown in Figure 6. It can be seen that the OMP sparse representation method has stable and reliable noise reduction ability and is obviously superior to IIR filtering.

**Figure 7:**
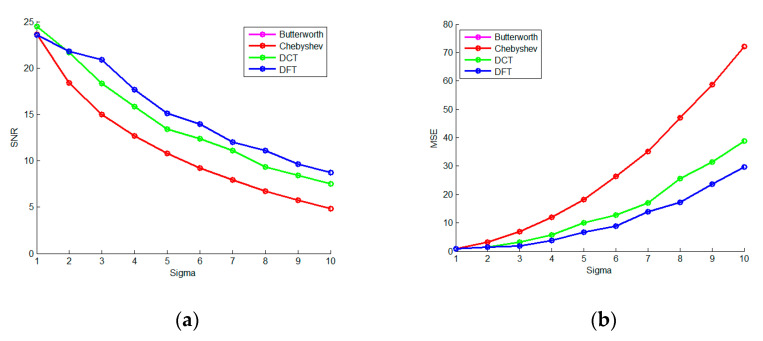
The Experiment on Intensity Change of Noise (**a**) is the SNR curve of four denoising methods when the sigma changes from 1 to 10, and (**b**) is the MSE curve of four denoising methods when the sigma changes from 1 to 10.

Change the sigma from 1 to 10 in sequence, as shown in Figure 7. It can be seen that the OMP sparse representation method based on the DFT dictionary has the best denoising effect and stable performance; the OMP sparse representation method based on the DCT dictionary has a modest effect, but it is relatively stable.

The traditional frequency-domain filtering method cannot remove the noise in the same frequency band. It is worth emphasizing that the deficiency is improved a lot in the sparse representation filtering method. At the same time, the model does not have the problem of phase lag.

### 4.3. Experiment of Denoising Methods Based on Compressive Sensing

Based on the relevant content in Section 2.2, this experiment takes DCT dictionaries as sparse dictionaries, and the Gaussian matrix, Bernoulli matrix, PartHadamard matrix, and SparseRandom matrix are respectively used as sampling matrices to carry out denoising experiments based on compressed sensing. The data length N is defined as 1024, and the BPDN algorithm is used in the reconstruction algorithm.

**Figure 8:**
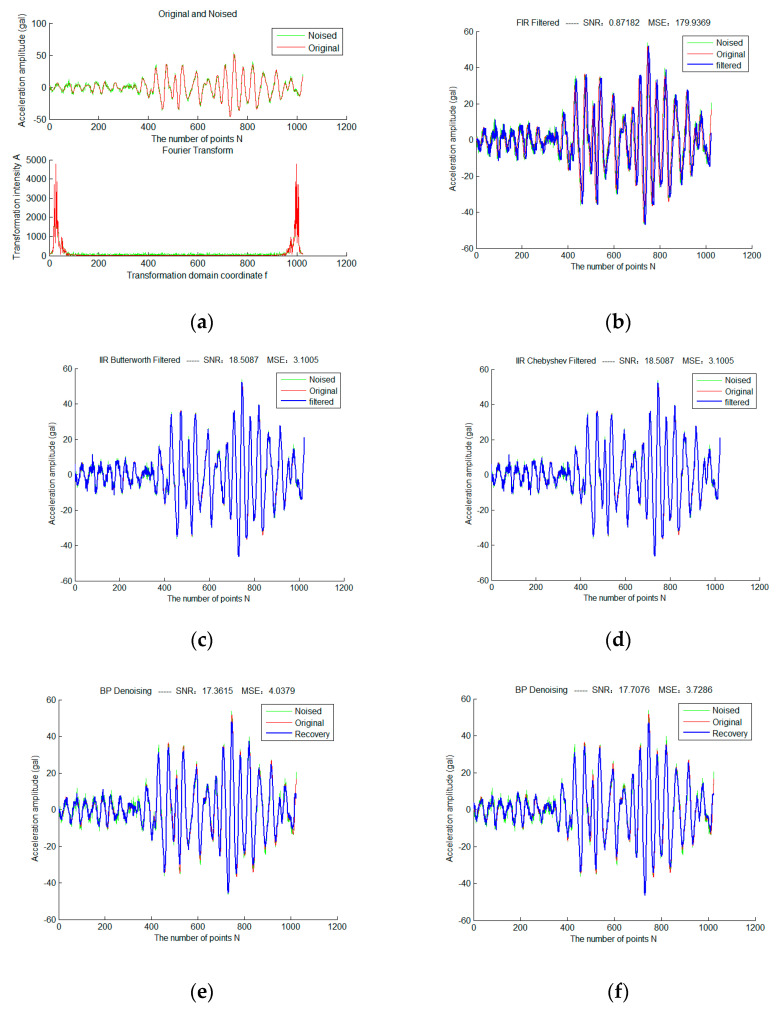

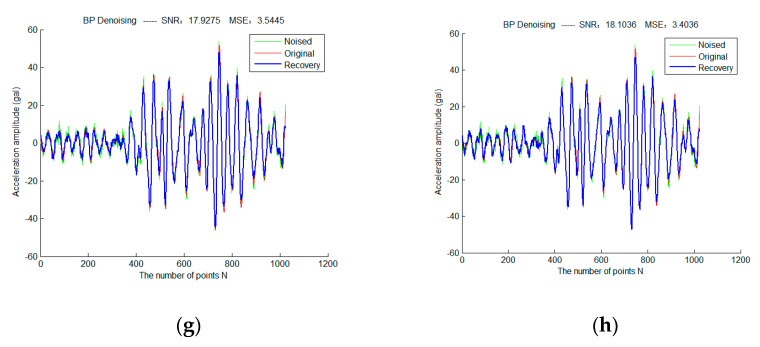
The Compressive Sensing Denoising Experiment of a Frame of Strong Earthquake Signals Based on Different Sampling Matrices (**a**) is the original and noise-adding waveforms of the frame signal in the time and frequency domain(sigma = 2); (**b**) is the result based on FIR filtering; (**c**) is the result based on Butterworth filtering; (**d**) is the result based on Chebyshev filtering; (**e**) is the BPDN compressive sensing denoising result based on the Gauss sampling matrix; (**f**) is the BPDN compressive sensing denoising result based on the Bernoulli sampling matrix; (**g**) is the BPDN compressive sensing denoising result based on the PartHadamard sampling matrix; (**h**) is the BPDN compressive sensing denoising result based on the SparseRandom sampling matrix.

First, the Gaussian white noise with sigma = 2 is added to the basic signal; Traditional filtering algorithms and compressed sensor denoising methods with different sampling matrices are used separately for experiments, as shown in Figure 8.

It can be seen that when sigma = 2, Butterworth and Chebyshev filters obtain exactly the same filtering results as traditional filtering methods, except that FIR filters have poor performance due to phase lag.

**Figure 9:**
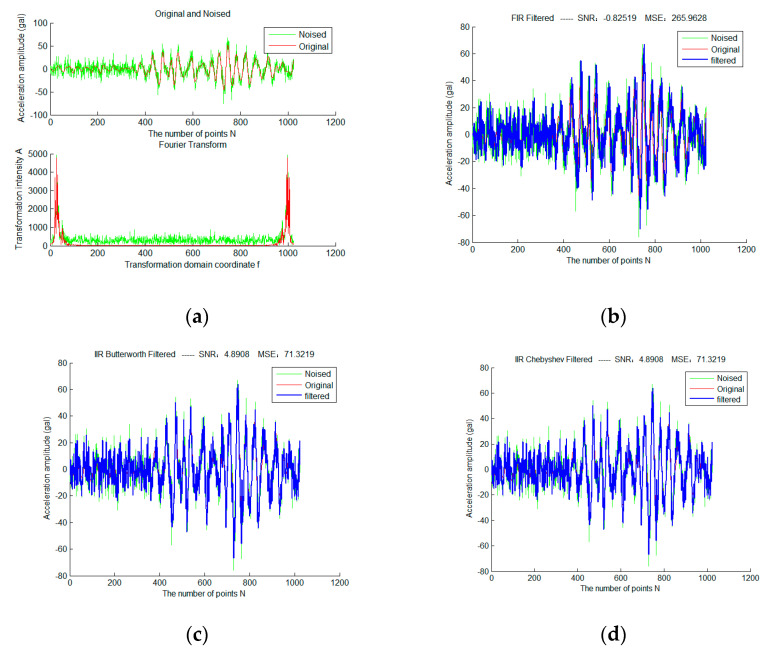

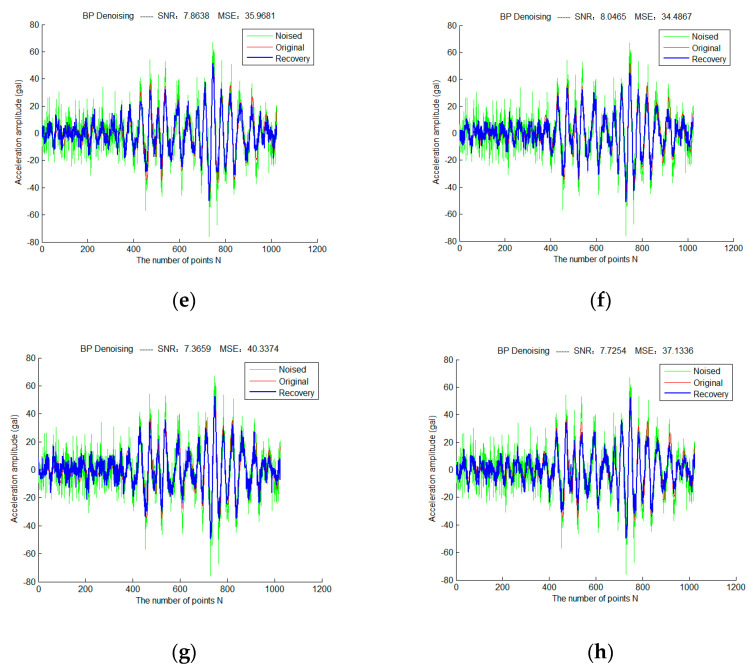
The Compressive Sensing Denoising Experiment of a Frame of Strong Earthquake Signals Based on Different Sampling Matrices (**a**) is the original and noise-adding waveforms of the frame signal in the time and frequency domain(sigma = 2); (**b**) is the result based on FIR filtering; (**c**) is the result based on Butterworth filtering; (**d**) is the result based on Chebyshev filtering; (**e**) is the BPDN compressive sensing denoising result based on the Gauss sampling matrix; (**f**) is the BPDN compressive sensing denoising result based on the Bernoulli sampling matrix; (**g**) is the BPDN compressive sensing denoising result based on the PartHadamard sampling matrix;(**h**) is the BPDN compressive sensing denoising result based on the SparseRandom sampling matrix.

Then, increase the sigma of the noise to 10, and repeat the above experiment. The result is shown in Figure 9.

It can be seen from Figure 9 that when the sigma of the noise is increased to 10, the effects of FIR filtering and BPDN compressive sensing methods are reduced together. However, the effects of the BPDN compressive sensing denoising method are still much better than that of traditional filtering.

**Figure 10:**
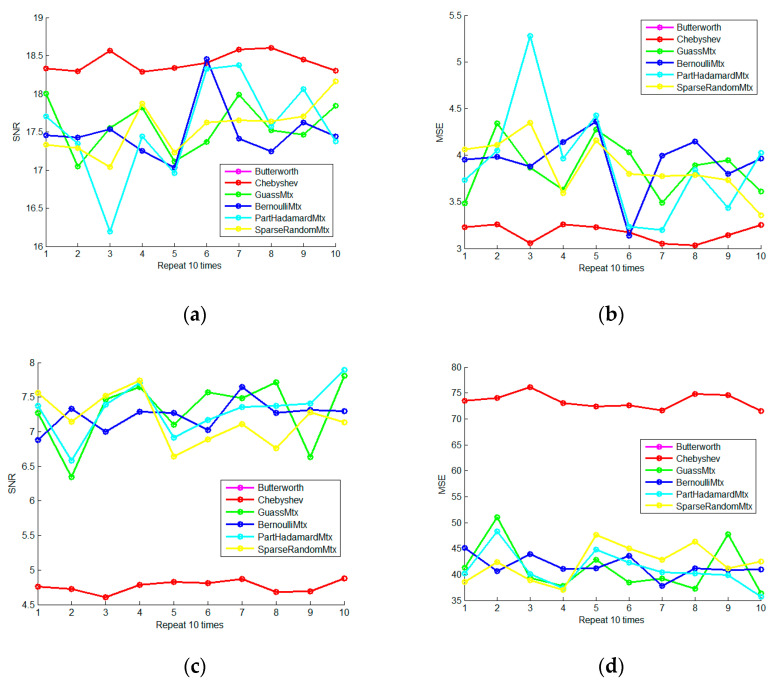
The Repeated Denoising Experiment (**a**) is the 10-times repetitive SNR curve of IIR filtering and BPDN compressive sensing denoising methods based on four sampling matrices when sigma is 2; (**b**) is the 10-times repetitive MSE curve of the denoising methods when sigma is 2; (**c**) is the 10-times repetitive SNR curve of the denoising methods when sigma is 10; (**d**) is the 10-times repetitive MSE curve of the denoising methods when sigma is 10.

In order to investigate the stability of the BPDN compressive sensing denoising method, we set the sigma to 2 and 10 and repeated the denoising experiment 10 times. The results are shown in Figure 10. It can be seen that because it can reduce the noise in the same frequency band as the signal, the BPDN compressed sensing denoising method is superior to the traditional filtering method. At the same time, this model does not have a phase lag problem similar to the traditional filter.

**Figure 11:**
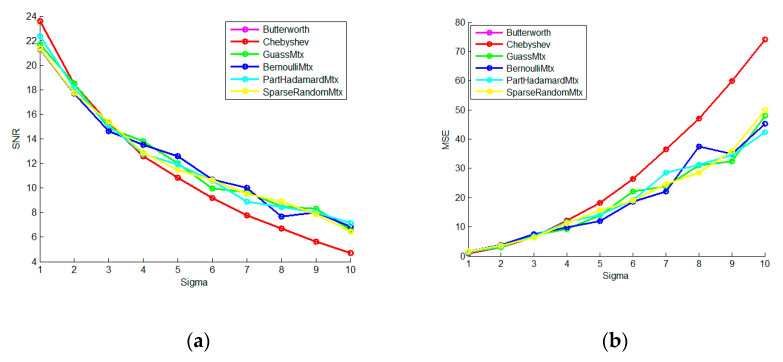
The Experiment on Intensity Change of Noise (**a**) is the SNR curve of FIR filtering and BPDN compressive sensing denoising methods based on five sampling matrices when sigma changes from 1 to 10, and (**b**) is the MSE curve of the denoising methods when sigma changes from 1 to 10.

Change the sigma from 1 to 10 in sequence, as shown in Figure 11. It can be seen when the sigma is greater than 3, the effects of the BPDN compressive sensing methods based on sampling matrices are always much better than that of the traditional filtering. When the sigma is less than or equal to 3, they are gradually worse than the FIR filtering. It means that the BPDN compressive sensing denoising method is suitable for reducing heavy noise.

### 4.4. Denoising Experiment Based on Learning-Type Dictionary

Based on the relevant content in Section 2.3, this experiment uses the KSVD algorithm to train the dictionary for spare denoising. For comparison, the data used in the experiment is the same basic signal as Section 4.2 and Section 4.3, and the data length N is 1024.

**Figure 12:**
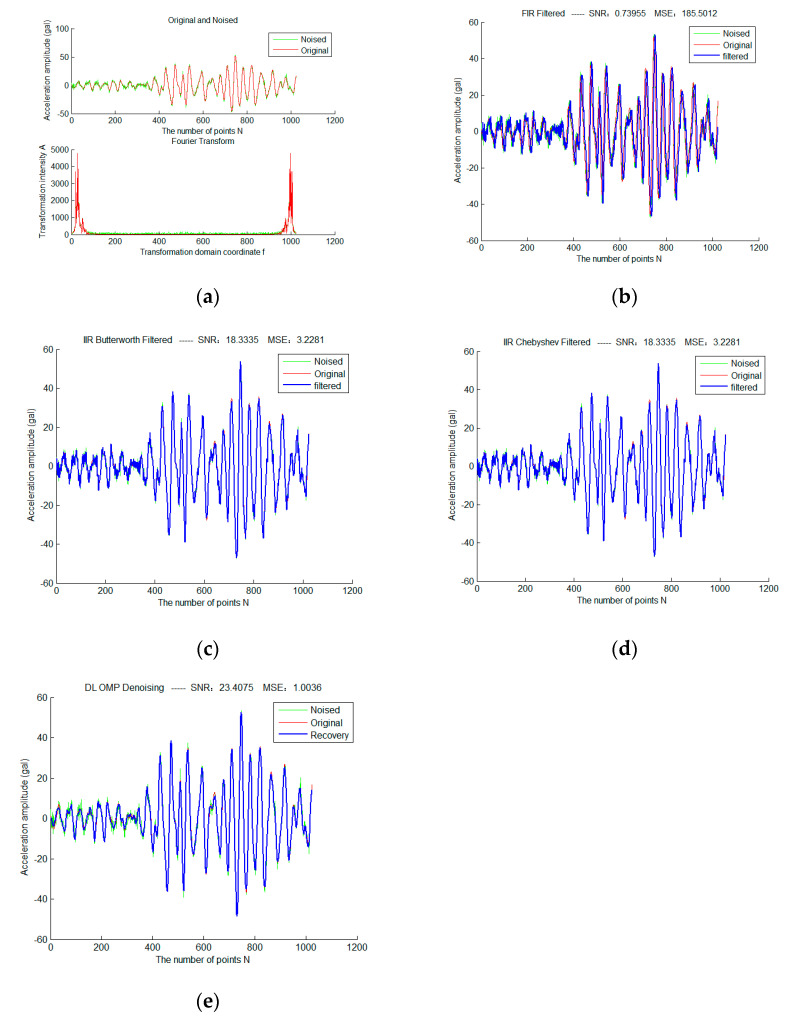
The Spare Denoising Experiment of a Frame of Strong Earthquake Signals Based on the Learning-type Dictionary (**a**) is the original and noise-adding waveforms of the frame signal in the time and frequency domain (sigma = 10); (**b**) is the result based on FIR filtering; (**c**) is the result based on Butterworth filtering; (**d**) is the result based on Chebyshev filtering; (**e**) is the OMP sparse representation denoising result based on the learning-type dictionary.

**Figure 13:**
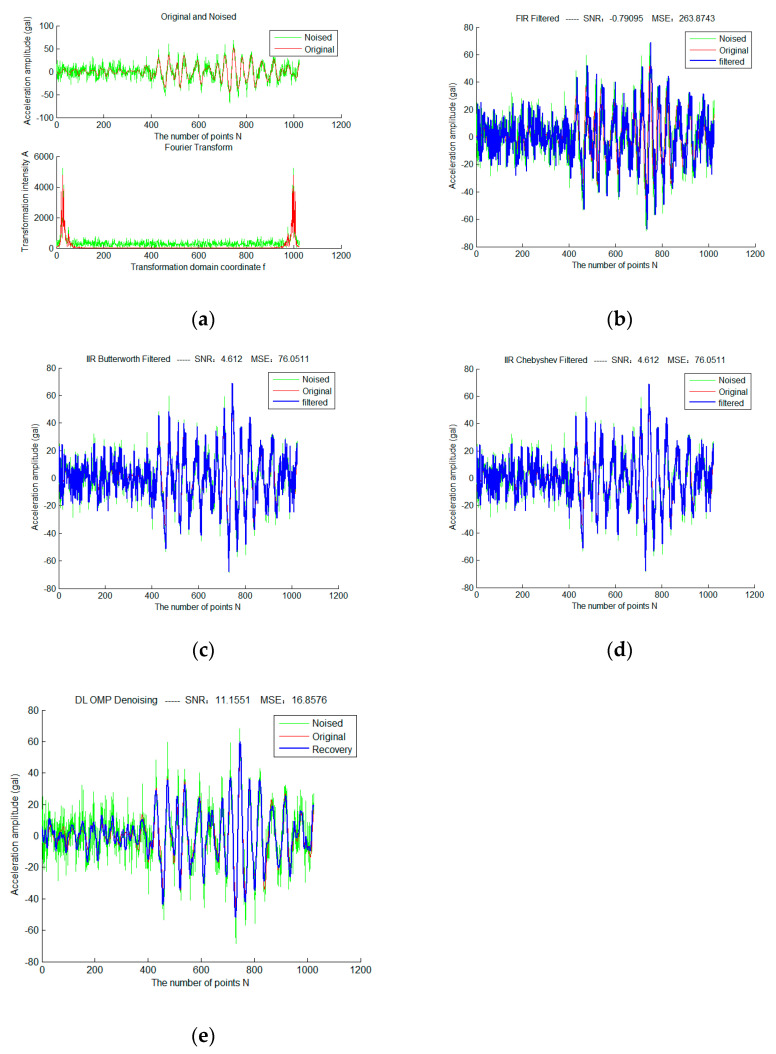
The Spare Denoising Experiment of a Frame of Strong Earthquake Signals Based on the Learning-type Dictionary (**a**) is the original and noise-adding waveforms of the frame signal in the time and frequency domain (sigma = 10); (**b**) is the result based on FIR filtering; (**c**) is the result based on Butterworth filtering; (**d**) is the result based on Chebyshev filtering; (**e**) is the OMP sparse representation denoising result based on the learning-type dictionary.

The advantage of the learning-type dictionary is that it can still obtain a better de-nosing result in the case of strong noise. In order to prove this point, Gaussian white noise (sigma is 2 and 10) is added to the specified basic signal. Traditional filtering and the method based on a learning-type dictionary are used for denoising experiments, respectively, which are shown in Figure 12 and Figure 13.

From Figure 12 and Figure 13, it can be seen that the spare denoising method based on the learning-type dictionary is better than traditional filtering, Whether the sigma is 2 or 10.

**Figure 14:**
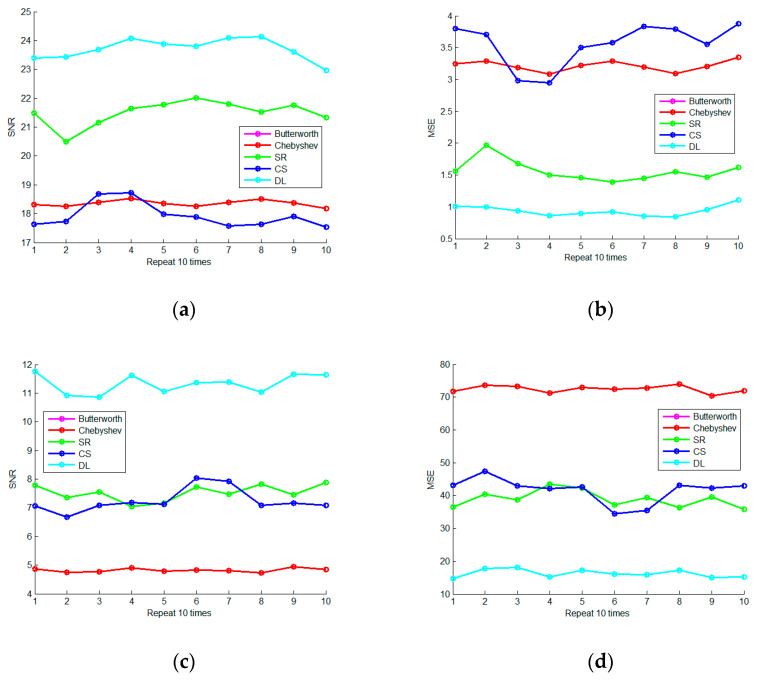
The Repeated Denoising Experiment (**a**) is the 10-times repetitive SNR curve of FIR filtering and three sparse methods when sigma is 2; (**b**) is the 10-times repetitive MSE curve of the denoising methods when sigma is 2; (**c**) is the 10-times repetitive SNR curve of the denoising methods when sigma is 10; (**d**) is the 10-times repetitive MSE curve of the denoising methods when sigma is 10.

In order to reveal the stability of the spare denoising method based on the learning-type dictionary, we set the sigma to 2 and 10 and respectively repeated the denoising experiments 10 times. The results are shown in Figure 14. Among them, SR refers to the OMP sparse representation denoising method, CS refers to the BPDN compressive sensing denoising method, and DL is an OMP denoising method based on the learning-type dictionary. It can be seen that the spare denoising method based on the learning-type dictionary is indeed optimal and stable in the scenarios of strong noise.

**Figure 15:**
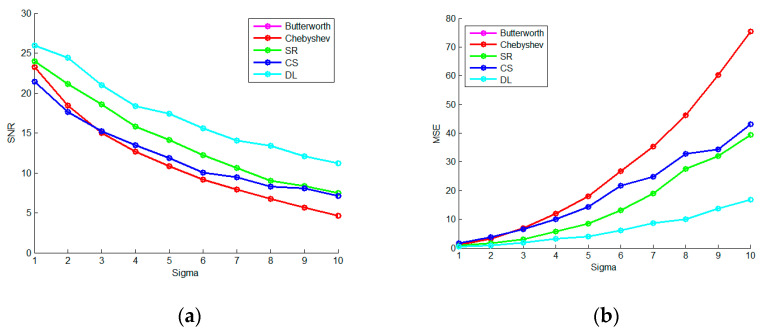
The Experiment on Intensity Change of Noise (**a**) is the SNR curve of the FIR filtering and three spare denoising methods when the sigma changes from 1 to 10, and (**b**) is the MSE curve of the denoising methods when the sigma changes from 1 to 10.

Change the sigma from 1 to 10 in turn, as shown in Figure 15. It proves again that the spare denoising method based on the learning-type dictionary is indeed better than other sparse and filtering denoising methods.

### 4.5. Experiment of Classifying Anti-Interference Method

Different from the full range denoising idea adopted in the previous sections, the classifying anti-interference method must satisfy the time efficiency request of earthquake early warning. In theory, it is necessary to make a classification judgment within three seconds of the first arrival of the P wave and remove noise interference to retain the real seismic waveform. Therefore, the length of the data used in this experiment was recorded at the first three seconds of the first arrival of the intercepted signal with the sampling rate of 200 sps, for a total of 600 points. There are nine major types of noise interference. In addition, with real seismic waveforms mixed, it adds up to 10 types, 1257 signals, of which 1005 are used for automatic learning and training, and the remaining 252 are used for testing, as shown in Table 1.

**Figure 16:**
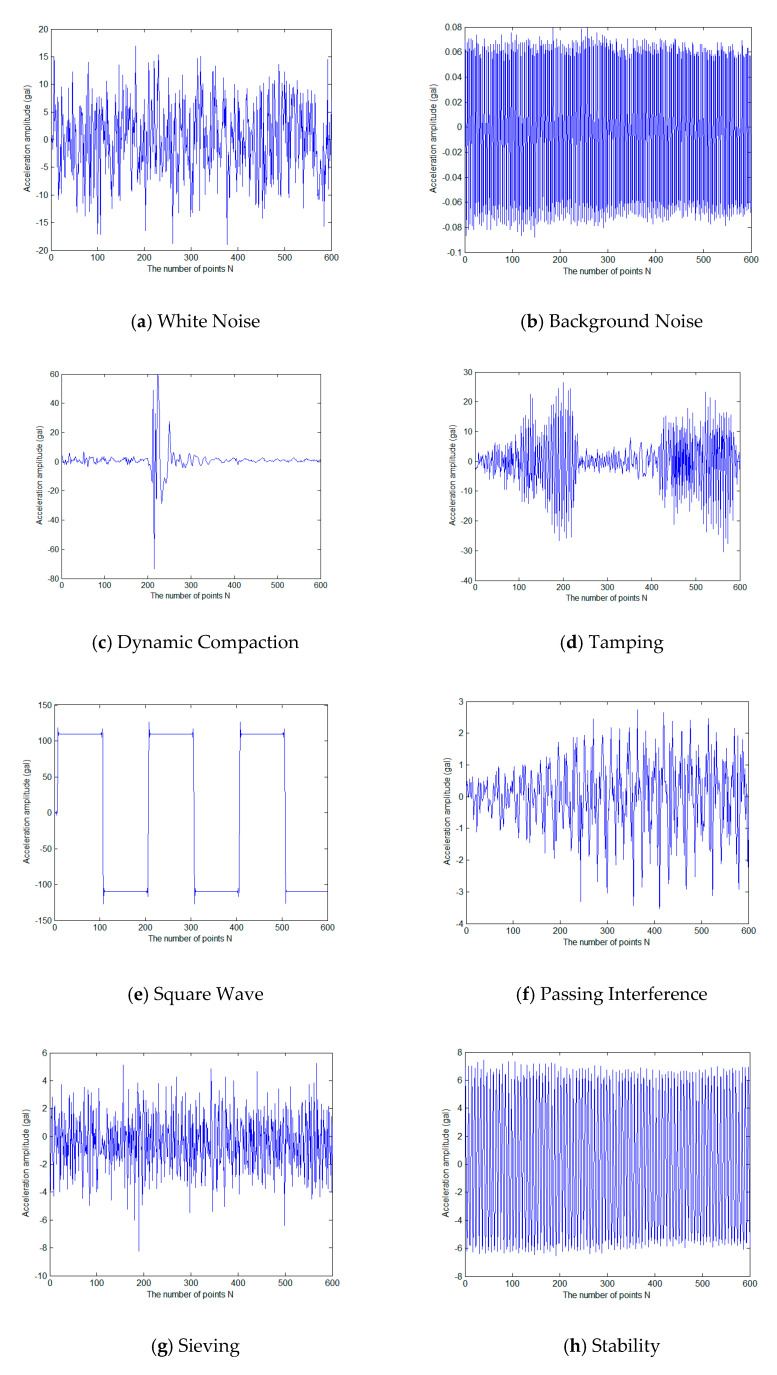

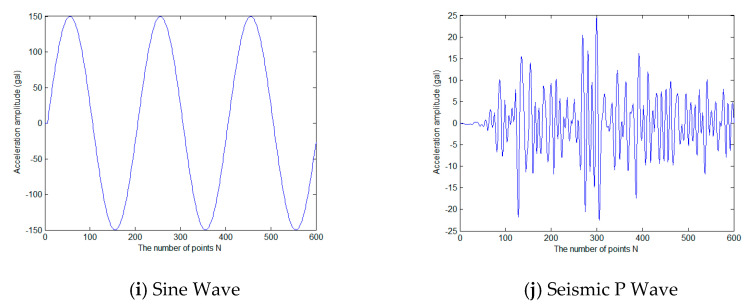
Examples of Various Types of Noise Interference Waveforms.

Among the nine types of noise, white noise, square wave, and sine wave are simulated data of interference signal, and the remaining six types of non-seismic interference are recorded by real strong motion seismometers at engineering sites. All types of various waveforms, including real seismic waveforms, are shown in Figure 16.

Based on the sparse method described in Section 3, the training set data are constructed into an over-complete dictionary, and the test set is sparsely classified. The accuracy of the classification is used to determine the effect of the algorithm. In order to ensure the reliability of experiments, all experiments are performed in the same environment. The experimental results of the classification accuracy are shown in Table 2.

From the experimental results, the accuracy rate of classifying the noise and real seismic waveform based on the selected test set reached 100%, and there were no false alarms for earthquake early warning. However, it is regrettable that the recognition rate of seismic waves is 84%, leading to some missed alarms. There is still a large space to develop. From the subdividing categories of noise, the recognition rates of the background noise, dynamic compaction, square wave, sieving, stability, and sine waves all reached 100%, while the recognition rates of white noise, tamping, and passing interference is relatively have large deviations. The passing interference is easily misjudged as white noise, background noise, tamping, or stability. It is certainly true that the classification results have a certain relationship with the number and proportion of samples. However, the collection of noise samples does have some difficulties, and it is difficult to make the number of each class the same.

### 4.6. Discussion

Based on the principles of sparse theories, the article elaborates on the relationship between three sparse representation theories, sparse representation, compressive sensing, dictionary learning, and denoising. The algorithm flow of denoising and the algorithm of dictionary learning are given. Performances of spare denoising methods are verified. It can be concluded that the spare representation denoising method based on a learning-type dictionary is of the best performance in the case of strong noise. The three sparse denoising models are better than the traditional filtering methods and improve the phase lag problem similar to FIR filtering.

Table 3 provides the comparison of the average SNR of three sparse denoising models and three traditional filtering methods under light noise (sigma = 2) and heavy noise (sigma = 10) (based on the experimental records in Section 4.2, Section 4.3, and Section 4.4).

Table 4 provides the comparison of average MSE of three sparse denoising models and three traditional filtering methods under light noise (sigma = 2) and heavy noise (sigma = 10) (based on the experimental records in Section 4.2, Section 4.3, and Section 4.4).

In Table 3 and Table 4, SR refers to the OMP sparse representation denoising method; CS refers to the BPDN compressive sensing denoising method, and DL is an OMP denoising method based on the learning-type dictionary.

In addition, for the interference waves of non-seismic events, this paper proposes an idea based on sparse representation classification to directly remove such non-seismic event interferences. From the experimental results, the accuracy rate of classifying the noise and real seismic waveform based on the selected test set reached 100%, which initially achieved the expected results.

**Figure 17:**
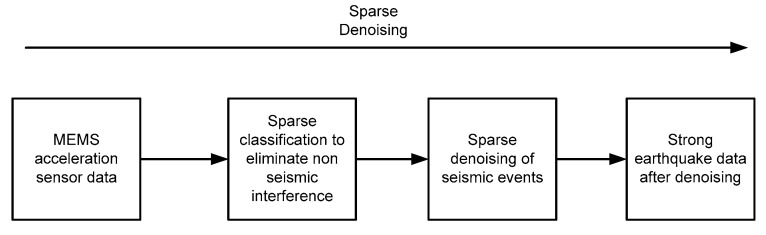
A standard sparse anti-interference denoising processes for strong earthquake early warning based on MEMS accelerometers.

According to the relevant content of this article, a standard sparse anti-interference denoising process for strong earthquake early warning based on MEMS accelerometers can be obtained, as shown in Figure 17.

## 5. Conclusions

Intensive seismic observation based on low-cost MEMS accelerometers effectively improves the acquisition speed of ground vibration information and the response speed of parameter output, which is expected to break through the blind spot of early warning while improving the timeliness of early warning. However, due to the presence of noise in strong ground motion data, the signal-to-noise ratio of the data is greatly reduced, which brings difficulties to the subsequent processing and also causes great interference with earthquake early warning. Because of the inherent defects of the traditional time-domain filtering method, it is not suitable for scenarios where the signal-to-noise ratio is too low, or the signal and noise are in the same frequency band. By aiming at this practical application problem, based on theories of sparse representation, compressive sensing, dictionary learning, and others, this paper deeply studies the sparse representation denoising problem of strong earthquake early warning based on MEMS accelerometers. Experiments show that the sparse representation denoising methods are effective. In addition, for the interference waves of non-seismic events, this paper proposes an idea based on sparse representation classification to remove such non-seismic interference directly.

At this stage, our work is mainly focused on theoretical methods. In the subsequent research, we will use these theoretical methods to test and verify the actual sensors. In practical applications, we need to face problems such as low-frequency noise (e.g., zero error of the sensors). Although these problems often have special processes to deal with in the MEMS system, we also need to consider these problems as a complete set of theories and solutions. In addition, in order not to increase the additional cost, the strong motion monitoring equipment based on MEMS accelerometers is usually sampled for simple ground installation or wall-mounted installation. It is also necessary to further study whether the specific occasions requiring the installation of auxiliary structural parts will bring further noise impact. Finally, the application of the sparse denoising model to earthquake early warning needs further exploration and research.

## Figures and Tables

**Table 1:** Type and quantity of experimental data.

Serial Number	Types	Total Number	Number of Training Sets	Number of Test Sets
1	White Noise	15	12	3
2	Background Noise	15	12	3
3	Dynamic Compaction	150	120	30
4	Tamping	18	15	3
5	Square Wave	39	30	9
6	Passing Interference	180	144	36
7	Sieving	21	18	3
8	Stability	21	18	3
9	Sine Wave	48	36	12
10	Seismic P Wave	750	600	150

**Table 2:** Experimental Results of Classification Accuracy.

Serial Number	Types	Number of Test Set	Number of Correct Results	Accuracy of Subclass	Category Accuracy
1	White Noise	3	0	0	Recognition rate of non seismic interference 100%
2	Background Noise	3	3	100%	
3	Dynamic Compaction	30	30	100%	
4	Tamping	3	2	66%	
5	Square Wave	9	9	100%	
6	Passing Interference	36	19	53%	
7	Sieving	3	3	100%	
8	Stability	3	3	100%	
9	Sine Wave	12	12	100%	
10	Seismic P Wave	150	126	84%	

**Table 3:** Comparison of SNR.

	SR	CS	DL	FIR	Butterworth	Chebyshev
Sigma = 2	21.96	17.77	23.75	0.87	18.41	18.41
Sigma = 10	8.73	7.29	11.07	−0.73	4.73	4.73

**Table 4:** Comparison of MSE.

	SR	CS	DL	FIR	Butterworth	Chebyshev
Sigma = 2	1.40	3.68	0.93	179.94	3.17	3.17
Sigma = 10	29.53	41.34	17.32	260.37	73.95	73.95

## Data Availability

The data used in the article is open in a limited time and can be accessed https://github.com/stsddking/jnx.
